# Supplementary figures and images for: In and Outs of Chuviridae Endogenous Viral Elements: Origin of a Potentially New Retrovirus and Signature of Ancient and Ongoing Arms Race in Mosquito Genomes
Source: Front Genet. 2020 Oct 22;11:542437. doi: 10.3389/fgene.2020.542437 (PMC7642597; doi:10.3389/fgene.2020.542437)

Tree scale: 0.01 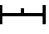

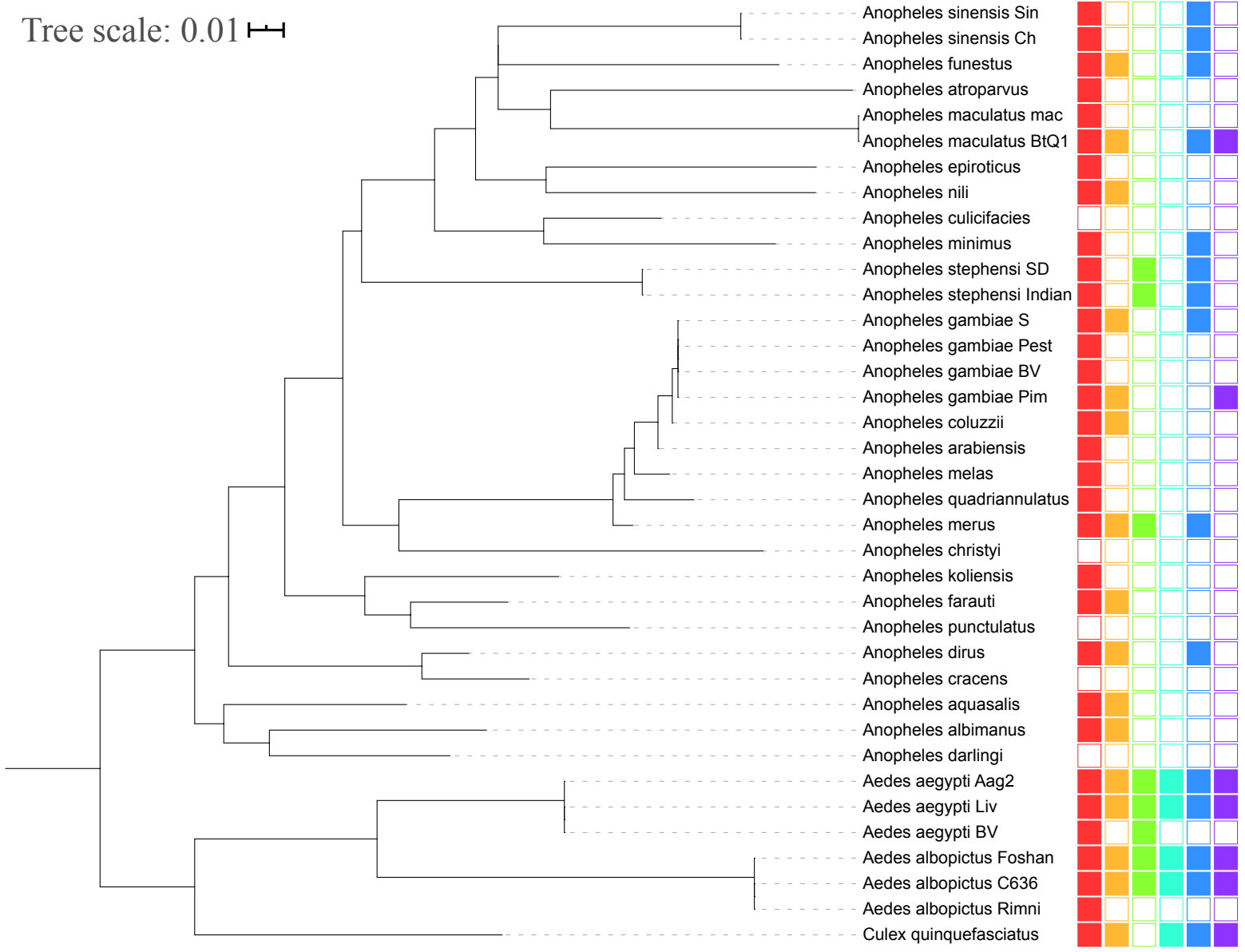

Supplement: Supplementary file 1 [file Data_Sheet_1.ZIP › Supplementary_Material_Anakin_MS_v2/Supplementary_Material_11.pdf]
